# *Candida parapsilosis* Colony Morphotype Forecasts Biofilm Formation of Clinical Isolates

**DOI:** 10.3390/jof7010033

**Published:** 2021-01-07

**Authors:** Emilia Gómez-Molero, Iker De-la-Pinta, Jordan Fernández-Pereira, Uwe Groß, Michael Weig, Guillermo Quindós, Piet W. J. de Groot, Oliver Bader

**Affiliations:** 1 Institute for Medical Microbiology, University Medical Center Göttingen, Kreuzbergring 57, 37075 Göttingen, Germany; emiliagomez803@hotmail.com (E.G.-M.); ugross@gwdg.de (U.G.); mweig@gwdg.de (M.W.); 2 Regional Center for Biomedical Research, Castilla-La Mancha Science & Technology Park, University of Castilla–La Mancha, 02008 Albacete, Spain; Jordan.Fernandez@alu.uclm.es; 3 Department of Immunology, Microbiology and Parasitology, School of Medicine and Nursing, Universidad del País Vasco (UPV/EHU), 48940 Bilbao, Spain; iker.delapinta@ehu.eus (I.D.-l.-P.); guillermo.quindos@ehu.eus (G.Q.)

**Keywords:** *Candida parapsilosis*, biofilm, colony morphology, drug susceptibility

## Abstract

*Candida parapsilosis* is a frequent cause of fungal bloodstream infections, especially in critically ill neonates or immunocompromised patients. Due to the formation of biofilms, the use of indwelling catheters and other medical devices increases the risk of infection and complicates treatment, as cells embedded in biofilms display reduced drug susceptibility. Therefore, biofilm formation may be a significant clinical parameter, guiding downstream therapeutic choices. Here, we phenotypically characterized 120 selected isolates out of a prospective collection of 215 clinical *C. parapsilosis* isolates, determining biofilm formation, major emerging colony morphotype, and antifungal drug susceptibility of the isolates and their biofilms. In our isolate set, increased biofilm formation capacity was independent of body site of isolation and not predictable using standard or modified European Committee on Antimicrobial Susceptibility Testing (EUCAST) drug susceptibility testing protocols. In contrast, biofilm formation was strongly correlated with the appearance of non-smooth colony morphotypes and invasiveness into agar plates. Our data suggest that the observation of non-smooth colony morphotypes in cultures of *C. parapsilosis* may help as an indicator to consider the initiation of anti-biofilm-active therapy, such as the switch from azole- to echinocandin- or polyene-based strategies, especially in case of infections by potent biofilm-forming strains.

## 1. Introduction

*Candida parapsilosis* was first described as a non-pathogenic yeast with no clinical relevance [1]. However, increased use of medical devices, parenteral nutrition, and nosocomial infections [2] has made *C. parapsilosis* one of the most critical fungal species causing blood stream infections (BSI) [3], which are of particular relevance in critically ill neonates [4,5,6] and immunocompromised patients [5,7].

The high infection rate with *C. parapsilosis* among neonates is likely due to the frequent requirement of parenteral nutrition [8] and the concomitant ability of *C. parapsilosis* to utilize fats and fatty acids as major energy sources [9]. In addition, the immature or compromised immune system may favor infections with this species [10].

Another risk factor for acquiring *C. parapsilosis* infections is the use of indwelling catheters and other medical devices onto which *C. parapsilosis* may form biofilms in conjunction with other *Candida* species or bacteria [4]. Primarily, this is attributed to its capacity to attach to the different materials of which medical devices are made [5,11,12]. This feature is highly variable among individual clinical isolates [13]. In *C. parapsilosis*, the formation of biofilms is associated with the ability to form pseudohyphae [14,15,16] as well as the concomitant change in expression levels of cell wall-localized adhesins such as Als1-7 [17,18,19] or Rbt1 [20]. Importantly, susceptibility to commonly used azole-based antifungal agents in fungal biofilms on medical devices may be strongly reduced [21,22].

**Figure 1:**
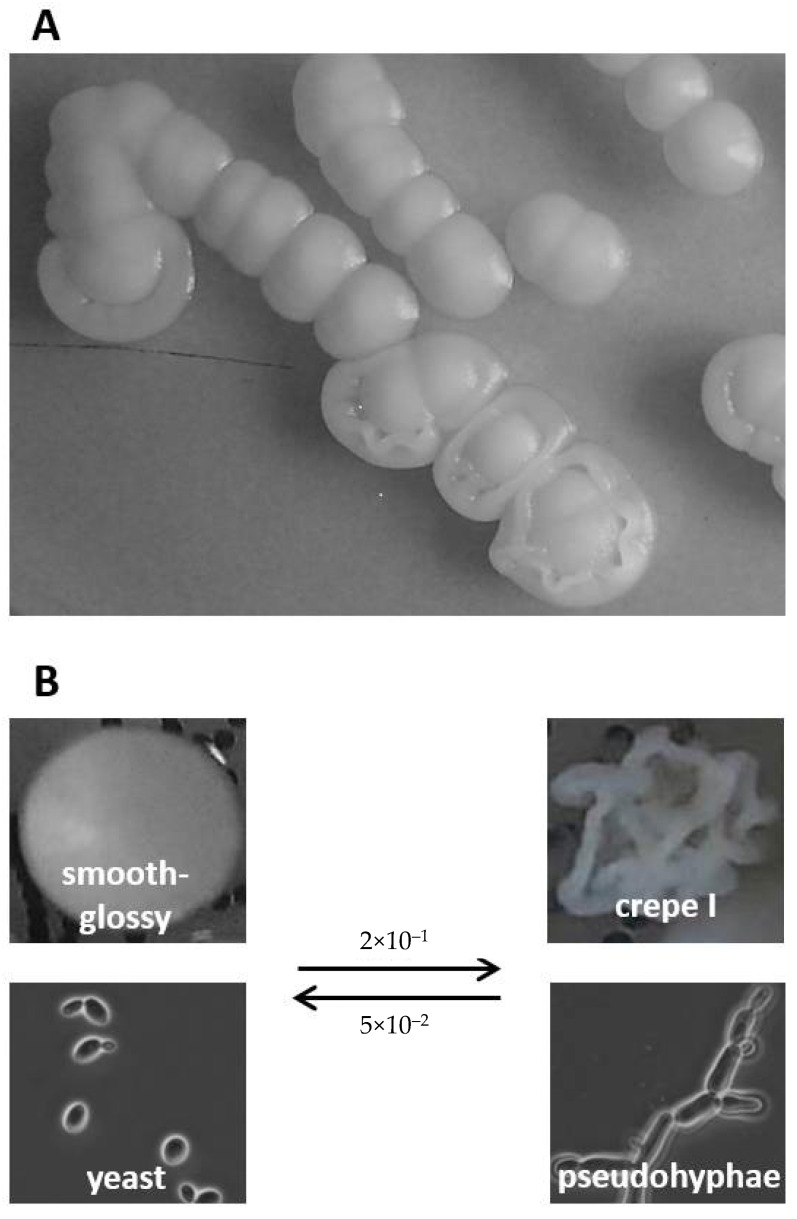
Colony morphotypes formed by *C. parapsilosis*. (**A**) Mixed morphotypes observed on a routine diagnostic plate. (**B**) Cells with distinct morphological colony phenotypes can be sub-cultured, but some strains (here strain PEU582: smooth-glossy vs. crepe, see below) sometimes undergo switching with strain-dependent frequencies. Smooth colonies are composed mainly of yeast-form cells, whereas non-smooth colonies are composed of pseudohyphal cells or mixtures of both morphologies.

Similar to *C. albicans* (reviewed in [23]), *C. parapsilosis* can have different colony morphotypes on diagnostic agar plates (Figure 1). While mixed-morphology culture plates can be the result of infection with multiple strains [24] in diagnostic procedures, also, the morphologic switching of some strains is a well-described phenomenon [25]. In addition to their role in biofilm formation, pseudohyphae formation and adhesin expression are also key to the visual appearance of fungal colonies on solid agars [26,27,28], which in turn may well be correlated to the capacity to form biofilms in the host, the incorporation of cell wall proteins (CWP), and, consequently, virulence [15].

Here, we phenotypically characterized a large collection of clinical *C. parapsilosis* isolates, including the description of novel intermediate morphotypes. We determined if early colony morphology was a potential predictor of biofilm production and pseudohyphal growth and as such might reveal the need to direct the antifungal therapy against biofilms containing *C. parapsilosis*.

## 2. Materials and Methods

### 2.1. Clinical Routine Diagnostic and Strain Maintenance

*C. parapsilosis* clinical isolates were routinely identified using MALDI-TOF (MALDI Biotyper, Bruker Daltonics, Bremen, Germany) according to the protocol described [29]. Mixed cultures were differentiated on YEPD agar (1% yeast extract, 2% peptone, 2% glucose, 2% agar) supplemented with 5 mg/mL phloxine B. On this medium, most tested colonies developed a final morphotype within 48 h of incubation at 30 °C (Figure 1, Supplementary Figures S1 and S2). Cells from colonies with stable morphotypes were transferred onto Sabouraud dextrose agar (SDA, Oxoid, Munich, Germany), regrown, and stored at −70 °C in cryovials (Mast Diagnostica, Reinfeld, Germany) for further analyses.

### 2.2. Biofilm Quantitation

Biofilm quantification assays on polystyrol were performed as described previously [11,30,31], with adaptations to suit *C. parapsilosis*. Briefly, isolates were grown on phloxine B-containing YEPD agar plates. Inoculum was prepared from single colonies grown to stationary phase in YEPD broth at 30 °C overnight at 220 rpm. A cell suspension adjusted to a cell density of McFarland = 2 was prepared using sterile saline, and 100 μL YEPD medium plus 50 μL aliquots of the cell suspensions were mixed in 96-well polystyrol microtiter plates (Greiner Bio-one) and incubated for 24 h at 37 °C. After removal of the medium by aspiration, the attached biofilms were washed once with 200 μL of distilled water. Cells were stained for 30 min in 100 μL of 0.1% aqueous crystal violet (CV). Excess CV was removed, and the biofilm was washed once with 200 μL of distilled water. To release CV from the cells, 200 μL of 1% SDS in 50% ethanol was added, and the cellular material was resuspended by pipetting. CV absorbance was quantified at 490 nm using a microtiter plate reader (MRX TC Revelation). Data shown are the average of three independent biological experiments, each including four technical repeats, using reference strain CDC317 as inter-experiment quality control.

### 2.3. Antifungal Drug Susceptibility Testing

Susceptibility testing was performed according to EUCAST e.def 7.3.1 standards [32]. Fluconazole (FLZ), voriconazole (VRZ), posaconazole (POS), and amphotericin B (AMB) substances were purchased from Discovery fine Chemicals Ltd. (Bournemouth, UK), micafungin (MFG) was kindly provided by Astellas, and caspofungin (CAS) was provided by Merck Sharp & Dohme Corp (MSD). Sequencing of the ERG11 and MRR1 genes was performed as previously described [33].

Preformed biofilms reduction

Cells were pre-grown on Sabouraud dextrose agar (SDA) for 96 h at 30 °C, and one colony was sub-cultured in 5 mL of YEPD broth overnight at 37 °C in an orbital incubator at 200 rpm. Cells were harvested by centrifugation and resuspended in Phosphate-buffered saline (PBS). Upon counting cells in a Neubauer chamber, the suspensions were adjusted to 1 × 10^6^ cells/mL in both RPMI (2 g/L glucose, pH 7) and YEPD (20 g/L glucose, pH 6.7). One hundred µL of the inoculum was pipetted into each well of a Bioscreen plate (Labsystem, Helsinki, Finland), and the plates were incubated for 24 h at 37 °C to allow biofilm formation. Next, planktonic cells were removed, and the plates were washed twice in PBS leaving just the biofilm in the wells. Two-fold serial dilutions of four antifungal drugs were prepared in RPMI or YEPD ranging from 0.25 to 32 µg/mL for POS, VRZ, and MFG, and from 0.0125 to 16 µg/mL for AMB. Subsequently, 100 µl of each dilution was added to the corresponding wells with biofilm in triplicate, and the plates were incubated again at 37 °C for 24 h. Finally, plates were washed twice with PBS, and reduction of the biofilm metabolic activity was determined by measuring the absorbance at 492 nm with the XTT (2,3-bis-(2-methoxy-4-nitro-5-sulfophenyl)-2H- tetrazolium-5-carboxanilide) colorimetric method [34].

### 2.4. Morphotype Development and Agar Invasion

Selected *C. parapsilosis* isolates were cultured overnight in YEPD liquid media in an orbital shaker at 220 rpm at 30 °C. Cell density was adjusted to 2 × 10^2^ cells/mL, after which 100 µL was plated onto YEPD agar plates and incubated at 30 °C for ten successive days. Starting after 48 h, the morphotype development of colonies was captured every 24 h over ten days using a stereoscopic binocular loupe (SZM-1, OPTIKA^®^, Ponteranica, Italy) mounted with a digital camera. Morphotypes and agar invasion were classified according to the references given in Supplementary Figure S1.

For analysis of agar invasion, colonies with different morphotypes were plated onto YEPD agar plates supplemented with 5 mg/mL of phloxine B [25], and morphotype development was followed as described above. Agar invasion was scored from day 4 onwards by scraping selected colonies with an inoculation loop and eventually washing off the cells under running water on the last day. Agar invasion was classified as low (1), low-medium (2), medium (3), medium-high (4), high (5), and finally as very high (6) when cells could not be removed by rinsing (see scoring reference in Supplementary Figure S1).

### 2.5. Statistical Analyses

For statistical analyses of biofilms and antifungal drug susceptibility, unpaired two-samples Student’s *t*-tests were used. All data used were the average of three independent analyses, and *p* values < 0.05 were considered statistically significant.

To detect potential correlations between biofilm formation capacity, colony morphotype, and/or agar invasion capacity, regression analyses were performed, and Pearson’s correlation coefficient r was used as a predictor for correlation. All data were analyzed using IMB SPSS 22 statistics software.

## 3. Results

### 3.1. Biofilm Formation Capacity Is Independent of Body Site of Isolation

**Figure 2:**
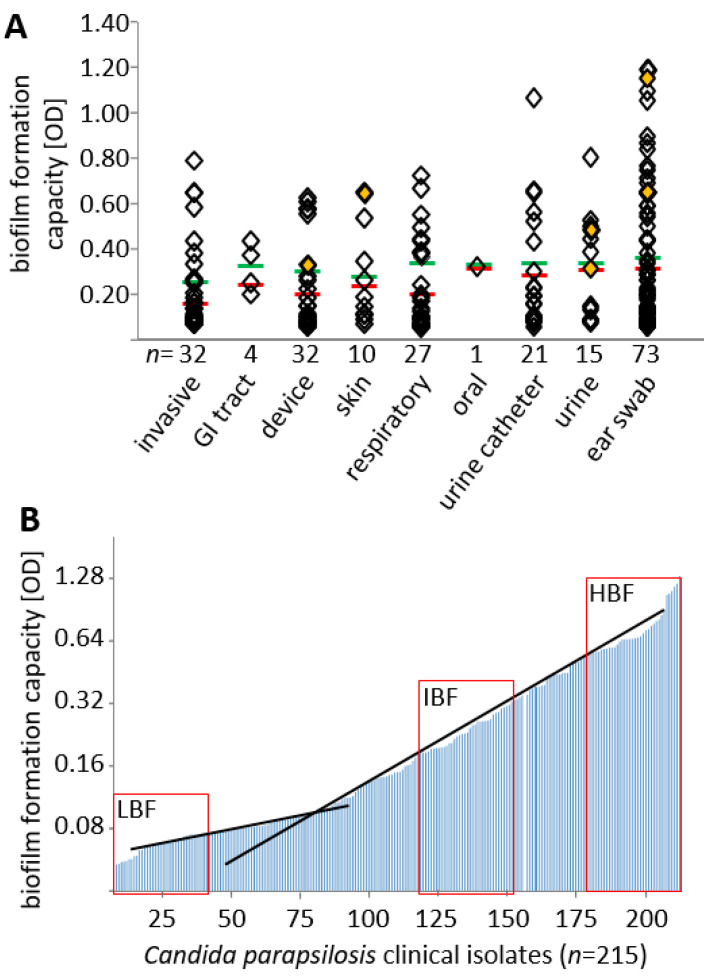
*C. parapsilosis* collection and selection of isolates for downstream experiments. (**A**) Distribution of collection isolates stratified according to site of isolation and biofilm production on polystyrol. Category “invasive” includes, e.g., blood culture, biopsies, or intraoperative swabs. Orange diamonds: six isolates used in pre-formed biofilm experiments, see text. Red and green lines: mean and two-fold mean values. (**B**) Biofilm formation capacity; isolates sorted by value from low to high. Intersection of black lines: approximated cut-off. Red boxes indicate strain selection of representative low (LBF, left box), intermediate (IBF, middle box) and high (HBF, right box) biofilm formers for subsequent experiments.

Over the course of two years, we collected 215 *C. parapsilosis* clinical isolates from our routine diagnostics (Figure 2). Isolates were classified according to nine different categories depending on the body site of isolation. *C. parapsilosis* is known to frequently occur in the nape region, reaching up to the ear. Consequently, most isolates stemmed from ear infections; however, a substantial number of isolates from invasive infections at other body sites as well as from indwelling devices such as central venous or urine catheters were included in the study. *C. parapsilosis* was only infrequently isolated from locations of the GI (2,3-bis-(2-methoxy-4-nitro-5-sulfophenyl)-2H- tetrazolium-5-carboxanilide) tract, the oral cavity, or the skin.

Isolates obtained were systematically screened for their capacity to form biofilms in standard biofilm formation tests in polystyrol microtiter plates. No body site, including those isolate groups obtained from plastic materials, could be identified to be significantly associated with elevated numbers of biofilm-forming isolates (*p* = 0.371, Figure 2A). When stratified by quantitative measurement values, we observed a near even distribution across the study group; only a tentative cut-off for low biofilm formation capacity was observed at OD (optical density) measurement values of approximately 0.1 (Figure 2B, intersection of black lines). Microscopical imaging of cells in biofilms from some representative isolates confirmed the already established idea that the capacity to form biofilms is correlated with pseudohyphal development (Supplementary Figure S3) [25,26,27,28].

### 3.2. Effect of Biofilm Formation on Antifungal Drug Susceptibility

In order to estimate the correlation of the lead phenotype (biofilm formation on polystyrol) with drug susceptibility, we selected 40 isolates each of low (LBF), intermediate (IBF), and high (HBF) biofilm formation capacity (Figure 2B, red boxes) from our collection including two non-adherent control strains (CDC317 and ATCC22019). The strains were tested for susceptibility to selected azoles (FLZ, POS, VRZ), echinocandins (CAS, MFG), and one polyene (AMB) after one (young colonies) and eight days (matured colonies) of growth on phloxine B agar plates.

Four IBF isolates (PEU651, PEU768, PEU941, and PEU950), and reference strain CDC317 showed elevated (minimum inhibitory concentration) MIC (values of 4–16 µg/mL for FLC. To exclude potential biases through resistance mutations (e.g., Y132F [35]), we sequenced the ERG11 and MRR1 genes in these isolates. A non-synonymous ERG11 point mutation was found only in CDC317, which was heterozygous with respect to the Y132F amino acid exchange. Y132F is known to confer resistance to fluconazole [36,37]. MRR1 only contained non-synonymous SNPs (Single nucleotide polymorphisms) in PEU651. Since we could not exclude a potential influence of these mutations, data for PEU651 as well as those of the two reference strains were excluded from the drug susceptibility analysis, leaving a total of 117 isolates in three groups of 39 isolates each.

**Figure 3:**
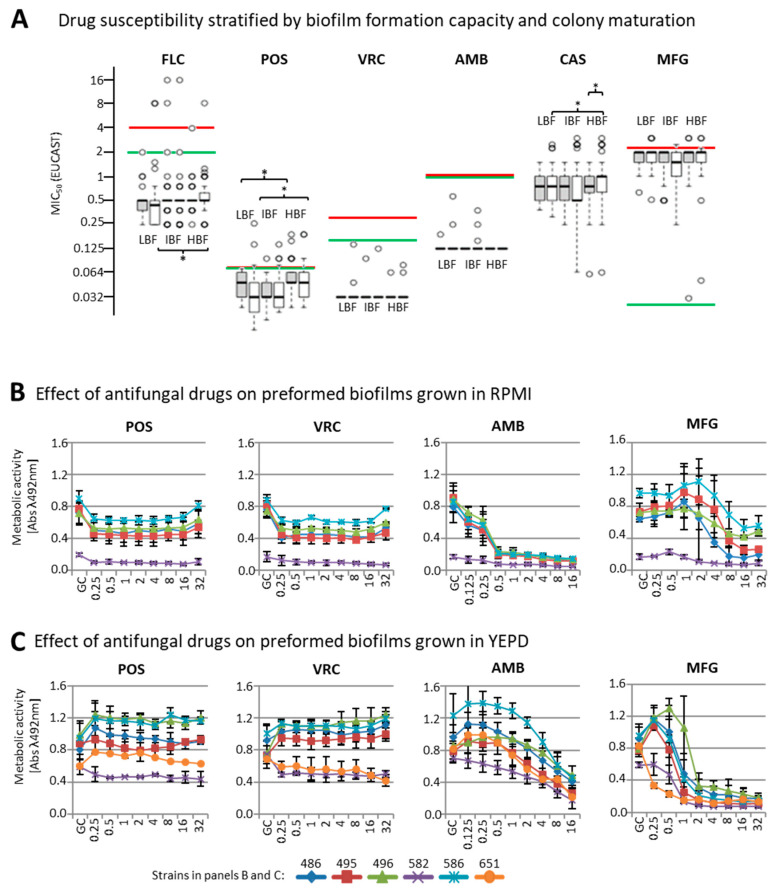
Drug susceptibility. (**A**) Biofilm formation-phenotype dependent susceptibility testing where inoculum was prepared from cells after 1 day of growth on Sabouraud dextrose agar (SDA) (gray boxes) and after 8 days of growth (white boxes) on the same plates, when colonies had fully developed morphotypes. For each substance tested, the values for 1 and 8-day inoculum are depicted for each group (LBF, IBF, and HBF). Red lines: EUCAST clinical breakpoint (R>); green lines, susceptible cut-off (S ≤). *: statistical significance (**B**,**C**) Effect of antifungal drugs on cell viability in pre-formed biofilms of selected biofilm-forming isolates tested in (**B**) RPMI (Roswell Park Memorial Institute )or (**C**) YEPD(yeast extract peptone dextrose) media.

We did not observe large-scale differences in drug susceptibility between experiments undertaken with young or mature colonies except for CAS (LBF *p* = 0.017 and HBF *p* = 0.028, Figure 3A white vs. gray boxes).

Although statistically significant differences between HBF versus IBF or LBF isolate groups were clearly evident for some antifungal drugs (POS: LBF vs. HBF with young colonies *p* = 0.014, mature colonies *p* = 0.072, CAS: LBF vs. HBF with young colonies *p* = 0.015), the observed mean MIC differences did not result in a major change in classification of either susceptible (S) or resistant (R) according to EUCAST breakpoints. Differences in mean susceptibility values were 1-2 log2-fold decreases for VRZ, POS (mature colonies only), CAS, and MFG in the HBF group, as compared to the LBF group. No apparent differences for either FLZ or AMB were seen.

### 3.3. Susceptibility of Biofilms to Antifungal Drugs

Next, for six selected intermediate to high biofilm-forming isolates, we analyzed to which degree pre-formed *C. parapsilosis* biofilms resisted antifungal drug treatment in two different media, RPMI and YEPD. RPMI is considered the reference medium for antifungal drug susceptibility testing and, as mentioned above, it was also used for MIC determination according to the EUCAST protocol. Likewise, YEPD is a glucose-rich medium that is widely used in assays with yeasts due to the large amount of peptone and dextrose extremely necessary for yeast growth and biofilm formation. The ability to form biofilms was evaluated using this culture medium. Since both media are widely used in the literature, we decided to test the drug susceptibility of preformed biofilms in both of them, observing that some strains are more prone to form biofilms in one media and not in the other, as well as behaving differently when interacting with antifungal drugs. More specifically, one of the six isolates (PEU651) was not able to form biofilm in RPMI (Figure 3B), whereas another (PEU582) grew only at reduced rates. Both isolates also showed a reduction in biofilm development in YEPD (Figure 3C) but were kept in these assays as the results were qualitatively in agreement with the other strains used.

Antifungal drugs had different quantitative effects when the assay was carried out in RPMI (Figure 3B) or in YEPD (Figure 3C). In YEPD, sub-inhibitory drug concentrations caused increases in metabolic activity, as measured by XTT reduction. This may be a stress-response effect countering drugs at these levels, and it was considered an artifact for the purpose of this study. Higher concentrations of azoles (POS, VRZ) reduced biofilm metabolic activity by only 30–50% as compared to the drug-free control. There was no azole drug concentration in the measurement range (up to 32 µg/mL) that led to a full reduction in biofilm metabolic activity (all *p* > 0.05). In contrast, both MFG and AMB did achieve a strong reduction of metabolic activity, although under different conditions: in RPMI, an AMB concentration of 0.5 µg/mL was sufficient for 70% reduction, while 16 µg/mL were required in YEPD. For MFG, this was the opposite: in YEPD, a clear effect was seen at 2 µg/mL with only residual metabolic activity apparent up to the upper assay boundaries (32 µg/mL).

### 3.4. Biofilm Formation Capacity on Polystyrol Correlates with Colony Morphotype and Agar Invasiveness

On culture plates, individual *C. parapsilosis* isolates showed specific, stable morphotypes. Only a minority of isolates (18%) were also able to switch between such morphotypes upon re-plating (Figure 1 and scoring reference shown in Supplementary Figure S1).

**Figure 4:**
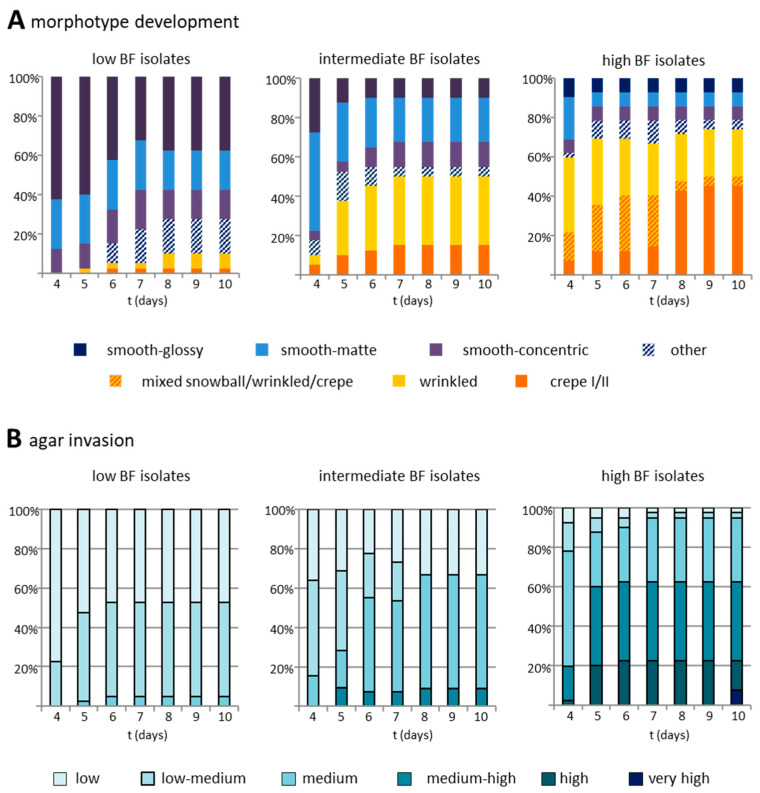
Emergence of colony morphotype over time. (**A**) Development of colony morphotype and (**B**) agar invasiveness scoring the same plates consecutively from 4 days to 10 days after inoculation. Isolates with low biofilm formation capacity (left panels), intermediate biofilm formation capacity (middle panels), and high biofilm formation capacity (right panels) were scored for the most frequent colony morphotype visible. Of note, HBF isolates rated “smooth” still developed minor frequencies of non-smooth colonies. See Supplementary Figure S1 for scoring references.

In a preliminary analysis on a selected subset (Supplementary Figure S2), non-smooth colony morphologies were already apparent at the start of the observation period (20%) and reliably appeared after 72 h of growth. Across the entire collection, agar invasion (Supplementary Figure S1A) and colony morphotype (Supplementary Figures S1B and S2) were therefore scored over a course of four to ten days (Figure 4). Most LBF isolates (90%) showed smooth morphotypes, and only a small proportion (10%), for instance PEU944, developed wrinkled or crepe phenotypes. On day 10, about 20% had developed non-smooth morphotypes as their major morphology (Figure 4A). With increasing biofilm formation capacity, also the frequency of non-smooth colony morphotypes increased. Of the IBF isolates, 20% had developed non-smooth morphotypes at day 4, and 55% had developed non-smooth morphotypes on day 10. HBF isolates mainly, but not exclusively, produced non-smooth phenotypes (66% on day 4, 83% on day 10), which in most cases were already distinguishable at day 2. Some isolates presented two independent stable morphotypes (e.g., PEU525: non-smooth (cr) and smooth (s)), which were distinguishable from day 2 until day 10. Along with the increased formation of non-smooth colony morphotypes in IBF and HBF strains (r = 0.832, *p* < 0.001), also agar invasiveness increased from day 4 to day 10 (r = 0.969, *** *p* < 0.001 (Figure 4B, Table 1).

## 4. Discussion

*C. parapsilosis* is frequently found as a cause of pathologies due to biofilm formation on medical devices in long-term hospitalized patients suffering from endocarditis, peritonitis, arthritis, or general sepsis [22,38,39,40]. We hypothesized that the capacity to form biofilm might be related to the origin of the clinical specimen, that is, infections at different body sites or fungus growing on medical devices such as indwelling catheters. However, when the 215 clinical isolates in our collection were scored for their biofilm-forming capacity on rich medium (YEPD), a strong inducer of biofilms in *C. parapsilosis* [41], no clear distribution of low (LBF) versus high (HBF) biofilm formation depending on the site of isolation was detected. Nevertheless, a high percentage of catheter-associated isolates belonged to the IBF and HBF groups, which is consistent with the notion that *C. parapsilosis* infections often start from infected indwelling devices [42,43].

Then, we raised the question of whether there might be a possible link between the biofilm-forming capacity of clinical isolates and their drug susceptibility [21,22], which could be useful for decision making about treatment strategies in the clinic. However, tests with the three groups of isolates (LBF, IBF, and HBF), reflecting a wide range—from negligible to high quantities of biomass—of biofilm-formation capacity on polystyrol, and six commonly used azole-, echinocandin-, and polyene antifungal agents did not reveal any such correlation. Observed MIC values were similar to those reported previously by others [44,45,46,47], and also, no remarkable differences were observed between inocula prepared from young or matured cells (as found in biofilms). Therefore, we conclude that the drug susceptibility data obtained with the standard EUCAST protocol do not seem to generate predictive information toward the biofilm-formation capacity of clinical isolates.

Nevertheless, fungal cells embedded within biofilms, including those formed by *C. parapsilosis*, are considered to be less susceptible to antifungal compounds [21,22]. This notion was confirmed here by studying the antifungal drug susceptibility of six biofilm-forming strains embedded in preformed biofilms, which showed significantly reduced sensitivity to azoles. This experiment was executed in both YEPD (high glucose) and RPMI (lower glucose) medium. As expected [41], biofilm formation in YEPD was higher than in RPMI, and media-dependent differences in echinocandin and polyene sensitivity were also noted. These observations support earlier reported overall quantitative dependences of biofilm formation and drug susceptibility on glucose levels in the media [41,48,49].

Another important aspect of our study was the question of whether *C. parapsilosis* colony morphotypes might perhaps serve to forecast the biofilm-forming capacity of clinical isolates. We hypothesized that high biofilm formers would show more non-smooth morphotypes and pseudohyphae than poor biofilm formers and have an increased tendency to invade agar [50]. When following colony morphotype development in the three isolate groups (LBF, IBF, and HBF) over a course of ten days, we indeed observed a strong correlation of the HBF group with non-smooth colony morphotypes. In contrast, the appearance of non-smooth colonies occurred only in a minority of the isolates in the LBF group. As surface adherence represents the first step in biofilm formation, our data are supported by studies showing that non-smooth colonies are generally more adherent to plastic than smooth colonies [51].

Interestingly, in most cases, non-smooth morphotypes could already be observed within 48 h of growth, and only little change was observed after 96 h, indicating that prolonged incubation beyond this time point is not needed for morphotype determination. In addition, all the morphotypes appeared stable, as they were reproduced upon repeating the experiment, which is consistent with the idea that switching is not a common or frequent event. Finally, biofilm-forming capacity and the appearance of non-smooth morphotypes and pseudohyphae were positively correlated to agar invasion. However, a large proportion of smooth colony types also displayed medium-strength agar invasion, indicating pseudohyphae formation at least at the base of the colony [52,53].

In summary, our experiments show that there is a strong correlation between colony morphotype and biofilm formation capacity in isolates from clinical samples and that this is not reflected in the results from standard antifungal drug susceptibility testing. Our data are the first to indicate that the observation of non-smooth colony morphotypes of *C. parapsilosis* may help to consider the initiation of anti-biofilm-active therapy. This may include antifungal lock therapy and shifting treatment from azole-based to echinocandin- or polyene-based strategies to eliminate biofilms from catheters [39]. However, due to the inherent ability of some strains to switch between morphotypes [25,26,27,28,54], the absence of such colonies should not be taken as an absolute indicator that biofilms do *not* exist in the patient.

## Figures and Tables

**Table 1:** Agar invasiveness and colony morphology.

Morphology ^a^	*n*	Agar Invasion ^a^					
		Low	Low-Medium	Medium	Medium-High	High	Very High
smooth	59	34%	27%	5%	32%	2%	0%
wrinkled	22	5%	9%	23%	50%	9%	5%
mixed/infrequent	16	0%	6%	6%	63%	19%	6%
crepe I and II	25	4%	4%	0%	36%	28%	28%

^a^ see Supplementary Figure S1 for classification of agar invasion and morphotypes.

## Data Availability

The data presented in this study are available upon reasonable request from the corresponding author.

## Supplementary Materials

The following are available online at https://www.mdpi.com/2309-608X/7/1/33/s1, Supplementary Figure S1. Morphological classification of *C. parapsilosis* colony morphotypes. (A) Semi-quantitative classification of agar invasion on phloxine B-containing SDA. (B) Colony morphotypes observed in our isolate collection. In picture pairs, left pictures represent colony morphotype after 10 d incubation, right pictures show agar imprint left on phloxine B agar plates after flushing off colonies with running water. Scale bars = 0.25 cm; Supplementary Figure S2. Colony morphotype development during a ten-day time-lapse experiment. Morphotype development of colonies was followed during ten days of growth on YEPD agar at 30 °C. (A) Five selected low biofilm-forming (LBF) clinical isolates, (B) five intermediate biofilm-forming (IBF) isolates, and (C) five high-biofilm-forming (HBF) isolates. d, derby; cn, concentric; cr, crater; s, smooth; wr, wrinkled; Supplementary Figure S3. Morphology of *C. parapsilosis* cells in biofilms onto polystyrol. Representative strains with low (LBF, row 1), intermediate (IBF, row2), and high (HBF, rows 3 and 4) biofilm formation capacity are shown. Biofilms were let to develop for 24 h in YEPD as described in materials and methods. Unbound cells were removed by washing with PBS. Remaining biofilm cells were observed with a Leica DM1000 microscope mounted with a HC PL 100×/1.32 objective and MC170 HD digital camera.
