# Supplementary material for: Candida parapsilosis Colony Morphotype Forecasts Biofilm Formation of Clinical Isolates
Source: J Fungi (Basel). 2021 Jan 7;7(1):33. doi: 10.3390/jof7010033 (PMC7827155; doi:10.3390/jof7010033)
Supplement: Supplementary file 1 [file jof-07-00033-s001.pdf]

Supplementary Figure S1

Classification references of *C. parapsilosis* colony morphotypes

A agar invasion appearance

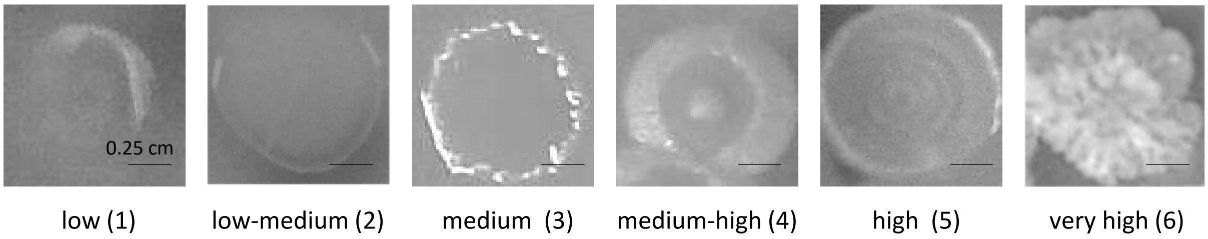

B colony morphotypes

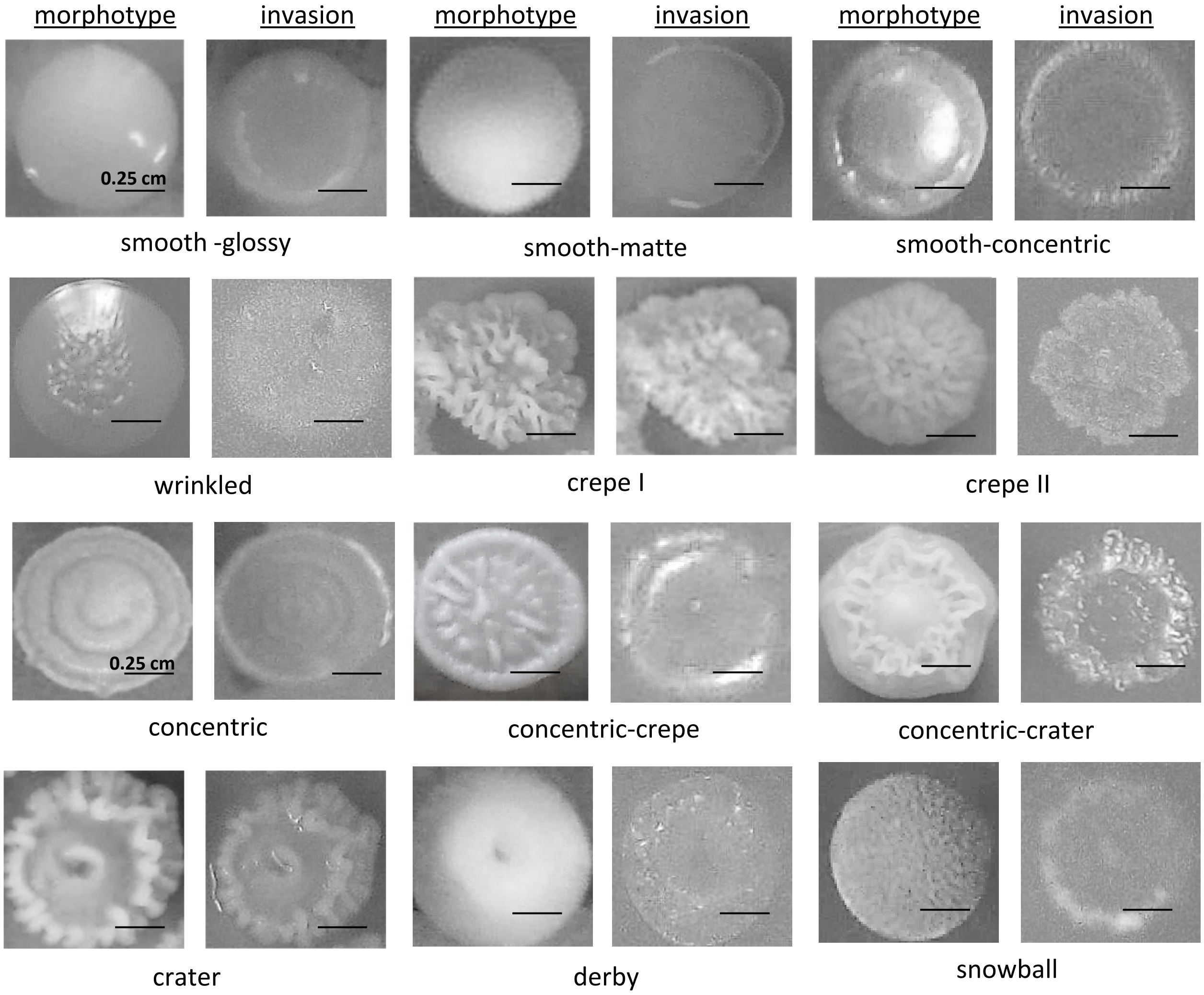

Supplementary Figure S1. Classification references of *C. parapsilosis* colony morphotypes. (A) Semi-quantitative classification of agar invasion on phloxine B-containing agar. (B) Colony morphotypes observed in our isolate collection. In picture pairs, left pictures represent colony morphotype after 96 h incubation, right pictures show agar imprint left on phloxine B agar plates after flushing off colonies with running water at day 10. Scale bars=0.25 cm.

Supplementary Figure S2A

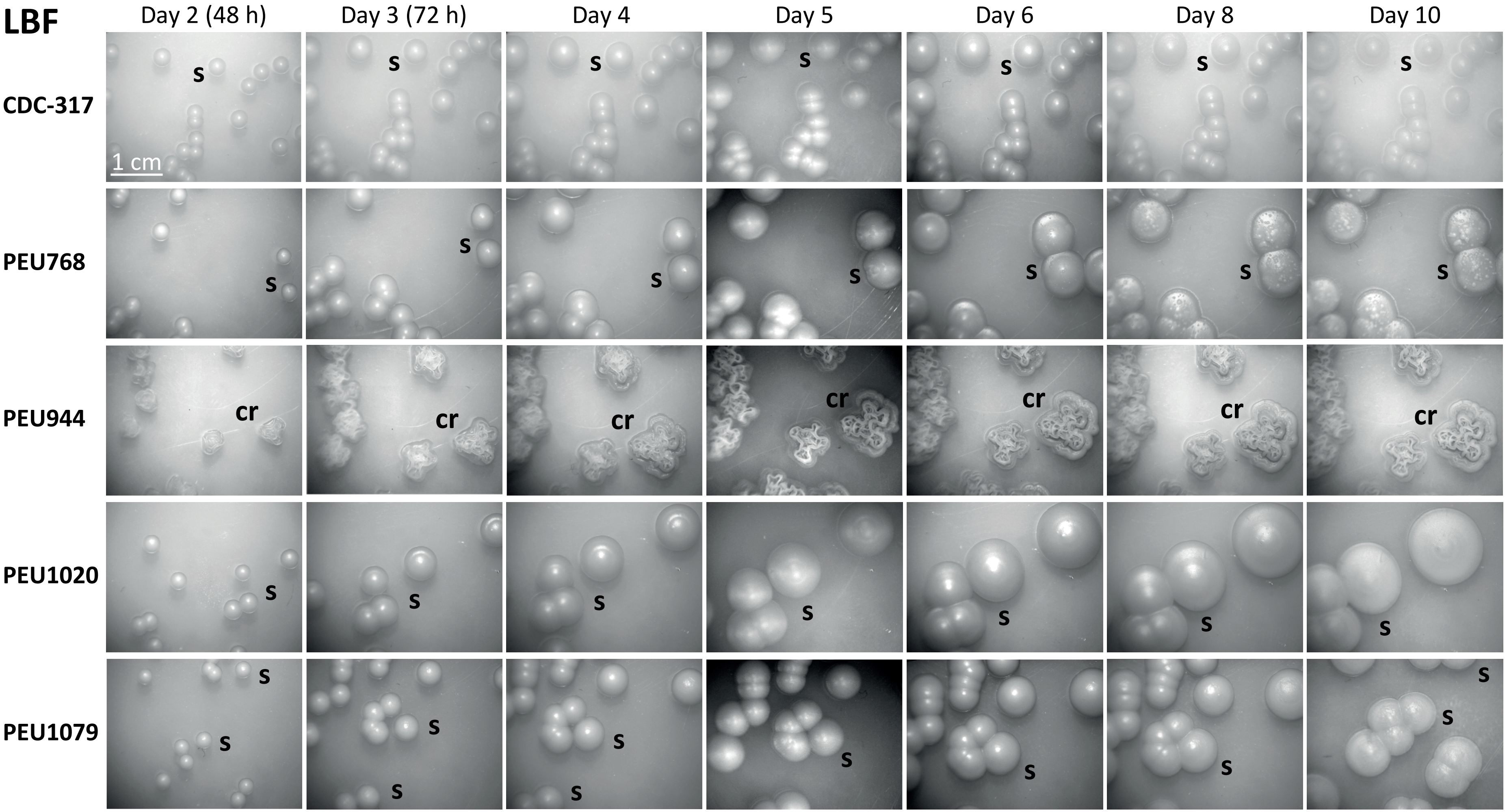

Supplementary Figure S2B

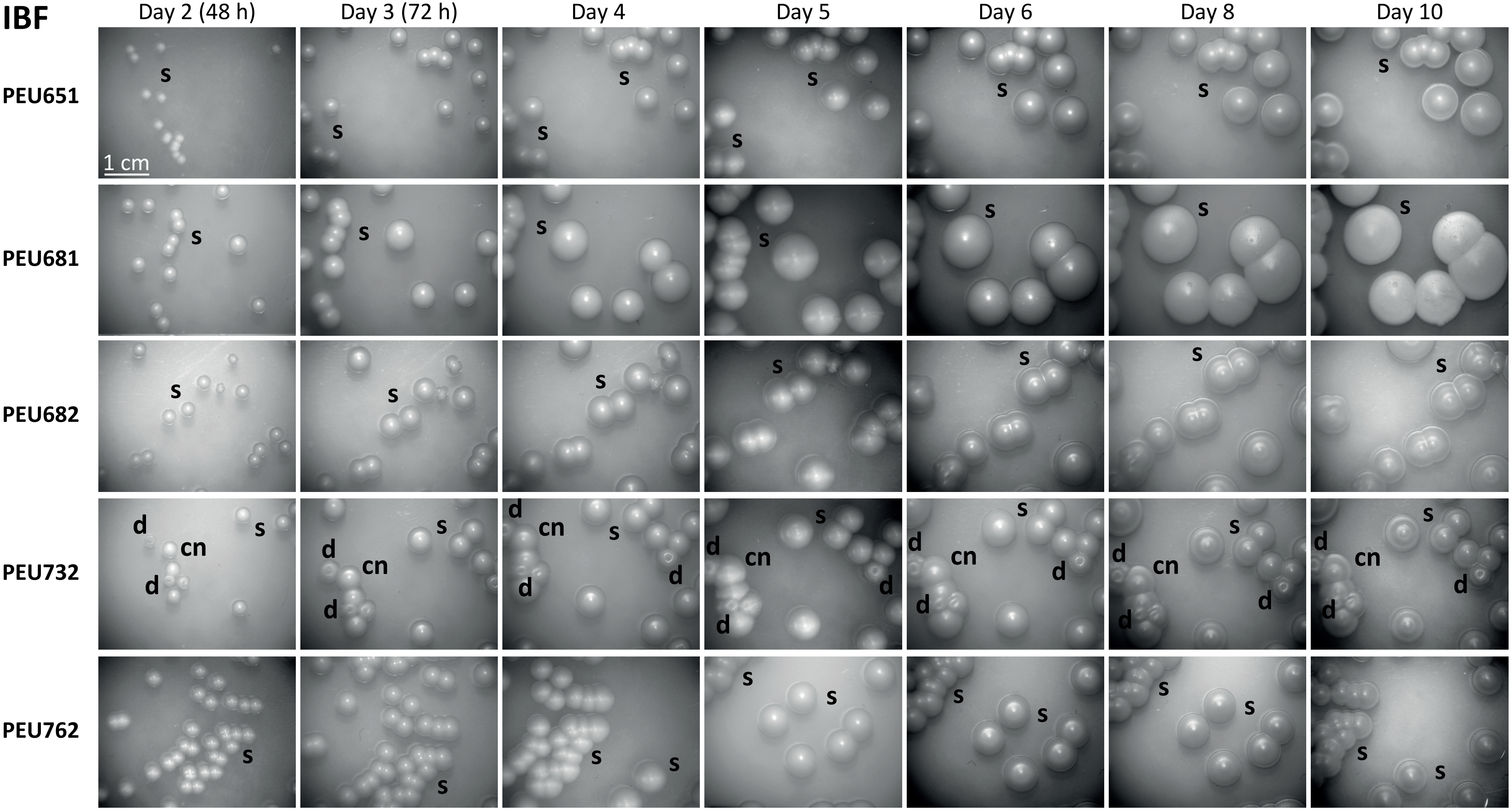

Supplementary Figure S2C

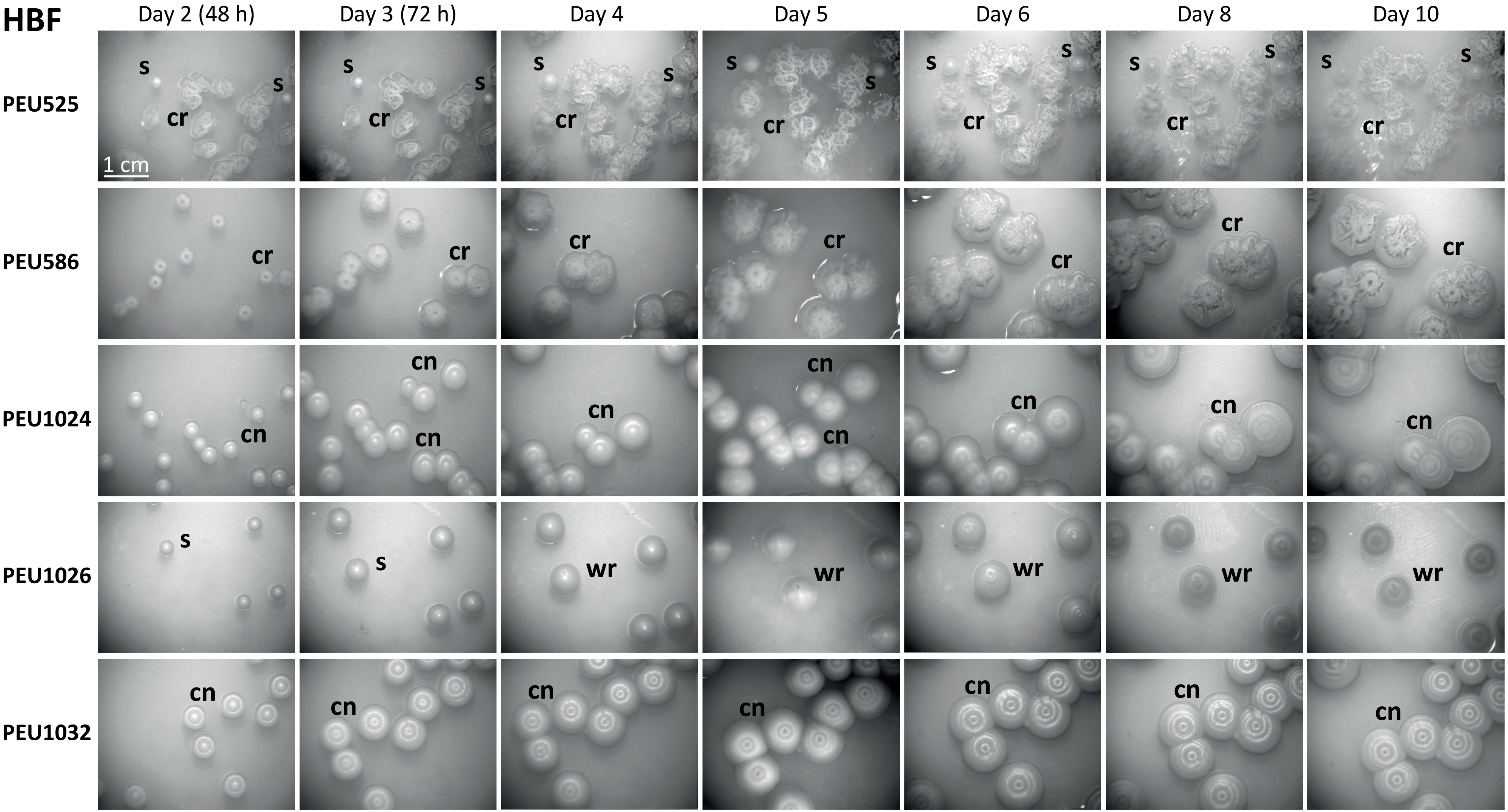

Supplementary Figure S2. Colony morphotype development during a ten-day time-lapse experiment. Morphotype development of colonies was followed during ten days of growth on YEPD agar at 30°C. (A) Five selected low biofilm-forming (LBF) clinical isolates,(B) five Intermediate biofilm-forming (IBF) isolates, and (C) five high-biofilm-forming (HBF) isolates. d, derby; cn, concentric; cr, crater; s, smooth; wr, wrinkled.

Supplementary Figure S3

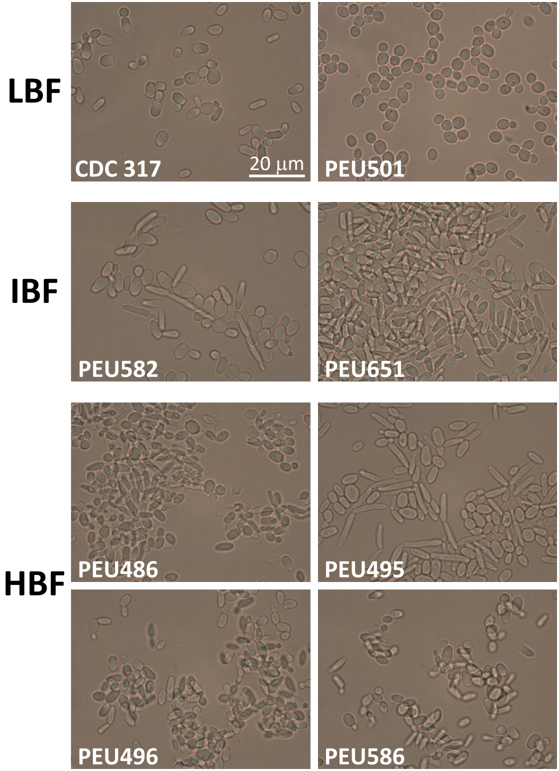

Supplementary Figure S3. Morphology of *C. parapsilosis* cells in biofilms onto polystyrol. Representative strains with low (LBF, row 1), intermediate (IBF, row2), and high (HBF, rows 3 and 4) biofilm formation capacity are shown. Biofilms were let to develop for 24 h in YEPD as described in materials & methods. Unbound cells were removed by washing with PBS. Remaining biofilm cells were observed with a Leica DM1000 microscope mounted with a HC PL 100x/1.32 objective and MC170 HD digital camera.
